# Developing guideline-based key performance indicators for recurrent miscarriage care: lessons from a multi-stage consensus process with a diverse stakeholder group

**DOI:** 10.1186/s40900-022-00355-9

**Published:** 2022-05-14

**Authors:** Marita Hennessy, Laura Linehan, Rebecca Dennehy, Declan Devane, Rachel Rice, Sarah Meaney, Keelin O’Donoghue

**Affiliations:** 1 Pregnancy Loss Research Group, Department of Obstetrics and Gynaecology, University College Cork, Cork, T12 DC4A Ireland; 2 INFANT Research Centre, University College Cork, Cork University Maternity Hospital, Cork, T12 DC4A Ireland; 3 College of Medicine and Health, University College Cork, Cork, T12 EKDO Ireland; 4 School of Nursing and Midwifery, National University of Ireland, Galway, Galway, H91 E3YV Ireland; 5 Evidence Synthesis Ireland, National University of Ireland, Galway, Galway, H91 E3YV Ireland; 6 School of Applied Social Studies, University College Cork, Cork, T12 D726 Ireland; 7 National Perinatal Epidemiology Centre, University College Cork, Cork University Maternity Hospital, Cork, T12 DC4A Ireland

**Keywords:** Early pregnancy loss, Care quality, Quality indicators, Quality improvement, Delphi technique, Patient and public involvement

## Abstract

**Background:**

Standardised care pathways tailored to women/couples who experience recurrent miscarriage are needed; however, clinical practice is inconsistent and poorly organised. In this paper, we outline our processes and experiences of developing guideline-based key performance indicators (KPIs) for recurrent miscarriage care with a diverse stakeholder group which will be used to evaluate national services. To date, such exercises have generally only involved clinicians, with the need for greater stakeholder involvement highlighted.

**Methods:**

Our study involved six stages: (i) identification and synthesis of recommendations for recurrent miscarriage care through a systematic review of clinical practice guidelines; (ii) a two-round modified e-Delphi survey with stakeholders to develop consensus on recommendations and outcomes; (iii) four virtual meetings to develop this consensus further; (iv) development of a list of candidate KPIs; (v) survey to achieve consensus on the final suite of KPIs and a (vi) virtual meeting to agree on the final set of KPIs. Through participatory methods, participants provided feedback on the process of KPI development.

**Results:**

From an initial list of 373 recommendations and 14 outcomes, 110 indicators were prioritised for inclusion in the final suite of KPIs: (i) structure of care (n = 20); (ii) counselling and supportive care (n = 7); (iii) investigations (n = 30); treatment (n = 34); outcomes (n = 19). Participants’ feedback on the process comprised three main themes: accessibility, richness in diversity, streamlining the development process.

**Conclusions:**

It is important and feasible to develop guideline-based KPIs with a diverse stakeholder group. One hundred and ten KPIs were prioritised for inclusion in a suite of guideline-based KPIs for recurrent miscarriage care. Insights into our experiences may help others undertaking similar projects, particularly those undertaken in the absence of a clinical guideline and/or involving a range of stakeholders.

**Supplementary Information:**

The online version contains supplementary material available at 10.1186/s40900-022-00355-9.

## Plain English summary

Women/couples who have recurrent/repeated miscarriages should receive care that meets their needs, through agreed care pathways. This is often not the case. Key performance indicators (KPIs) are measures of specific elements of care (structures, processes and/or outcomes), which can help us to judge the quality of care given. In this paper, we describe how we worked with women and men with lived experience, doctors, nurses, managers, and others, to develop and agree on a list KPIs for recurrent miscarriage care in Ireland. We will use these to check what services are doing across the country and what could be done better. Participants filled out surveys and took part in meetings to vote on and agree on what KPIs were important to include. They also shared their views and experiences of taking part in this work. Together, we developed 110 KPIs for recurrent miscarriage care. These include measures of how care is structured, counselling and supports, investigations and treatments provided, and health-related outcomes. Participants’ valued the different views that people brought to discussions and what they learned. They suggested ways that the process could be made more participant-friendly. For example, being up-front about the time it would take, explaining medical terms more, and cutting down on the number of items to be rated in surveys. It is important and possible to develop KPIs with different groups, particularly those with lived experience. Learning from our study may help others who want to do similar projects, such as develop KPIs or guidelines for care.

**Supplementary Information:**

The online version contains supplementary material available at 10.1186/s40900-022-00355-9.

## Background

The population prevalence of recurrent miscarriage is 1–3%, depending on the definition used, i.e. two miscarriages, or three or more miscarriages [1]. There are uncertainties around how to organise recurrent miscarriage care, including debates on the investigations and treatments that should be provided [2, 3]. While there are recurrent miscarriage guidelines [2, 3], clinical practice is inconsistent and poorly organised [2]. Women/couples often attend many different health care professionals/clinics searching for a cause and treatment [2, 4].

Three broad approaches to recurrent miscarriage care globally have been identified: (i) women receive minimal or no care until they have had three miscarriages when they then get investigated, (ii) graded approach through first and subsequent miscarriages, and (iii) women seen in a medical consultant-led clinic after two consecutive miscarriages and offered a full panel of investigations [2]. Standardised care pathways tailored to the need of women/couples instead of the current fragmented approach are required [2]. Recurrent miscarriage is often managed outside of clinical guidance, with suggested reasons including variances in definitions, poor quality evidence, and the strong desire for active management from women with recurrent miscarriage [5, 6]. While a minimum service for couples with recurrent miscarriage is needed globally, country-specific models of recurrent miscarriage care can vary according to healthcare system structures, opportunities for service development/reorganisation, and available resources [2]. While there are European guidelines [7], and others internationally [3], there is currently no clinical guideline for recurrent miscarriage care in Ireland, nor has the current provision of recurrent miscarriage services within all 19 maternity units/hospitals been examined.

Key performance indicators (KPIs) are specific and measurable elements of health and social care, based on standards determined through evidence-based academic literature and/or through the consensus of experts, which can be used to assess care quality [8, 9]. Categories of indicators include structure (i.e. context in which care occurs, as well as how care is organised), process (i.e. transactions between patients and providers throughout the delivery of healthcare), and outcome (i.e. effects of healthcare on the health status of patients and populations) [10]. KPIs can be developed from clinical practice guidelines [11, 12]; however, there is currently no gold standard approach to guideline-based KPI development [13, 14]. As clinical practice guidelines aim to improve quality-of-care processes, guideline-based KPIs predominantly relate to process quality [13]. Indicators relating to the process and structure of care provide specific areas for improvement, whereas good outcomes do not necessarily equate with good care quality given the multiple influencing factors [15–18].

Researchers in the Netherlands have previously developed guideline-based indicators for recurrent miscarriage care [18], and more recently, for Early Pregnancy Assessment Units [19]. Using the RAND-modified Delphi method, van den Boogard and colleagues developed 23 KPIs for care in couples with recurrent miscarriage from the 39 recommendations in the Dutch recurrent miscarriage guideline: These were all process indicators; no structural or outcome indicators were identified/included [18]. Furthermore, they included recommendations from only one guideline and did not involve those who experience recurrent miscarriage, only the expert opinions of 11 gynaecologists.

Lack of patient involvement is an identified limitation within the literature on guideline-based KPI development [13, 20] and guideline development more broadly [3, 21, 22]. In the development of guideline-based KPIs for UK primary care, despite some initial concerns, Rushforth and colleagues found that patient representatives were able to rate complex recommendations and gave similar opinions on feasibility, control and cost-saving criteria as health professionals on the panel [17]. Patients bring important lived experience insights; they also have a right, and should be given the opportunity, to contribute their views on outcomes, quality targets, and health care priorities that are important to them and to be actively involved in the development of KPIs [20]. While there is much-published guidance on how to develop clinical guidelines and guideline-based KPIs, few examples describe this process, particularly around how to include the views and priorities of diverse stakeholders, including health professionals, decision-makers and those with lived experience [23].

In this paper, we outline the process of developing KPIs for the investigation, management and follow-up of recurrent miscarriage, with a diverse stakeholder group, based on 32 clinical practice guidelines from high-income countries identified from a recent systematic review [3]. We define a stakeholder as “an individual or group who is responsible for or affected by health- and healthcare-related decisions” [24]. We share insights into our process experiences that may help others undertaking similar projects develop guideline-based KPIs.

## Methods

We conducted this work according to a pre-specified protocol (unpublished; deviations from protocol noted before the discussion section), which was developed utilising the Guidelines International Network Performance Measures Working Group reporting standards for guideline-based performance measure development and re-evaluation [14]. Patient and public involvement in this study is reported according to the GRIPP2-SF [25], available in Additional file 1.

### Composition of the guideline-based indicator development panel

This work was undertaken as an involvement activity with members of the RE:CURRENT Research Advisory Group (RRAG) to generate indicators to be used within a service evaluation. MH, RD, SM, LL, RR, DD and KOD conducted the systematic review of clinical practice guidelines [3] and led the KPI development process. The RRAG comprises 22 individuals with clinical, methodological and lived experience: healthcare and allied health professionals, representatives from advocacy and support organisations, those involved in the administration, governance and management of maternity services, academics, and women and men who have experienced recurrent miscarriage.

#### Ethical considerations

Ethical approval was not required for this project as it was an involvement activity with members of the RRAG to generate indicators that would be used within a service evaluation [26, 27]; this was confirmed by the Clinical Research Ethics Committee of the Cork Teaching Hospitals (Personal Communication 29/09/2020). Participation in the consensus activities was voluntary. The RRAG operates under an agreed Terms of Reference, including the following principles: respecting differences; no pressure to speak; listening; confidentiality. Members are also encouraged to contact the facilitators if they have any queries or if any issues arise for them. The research team was conscious that Group members brought different areas of expertise, for example, clinical or lived experience. Prior to the study commencing, in September 2020 at a regular meeting of the RRAG, we provided members with an introduction to KPI development (including what KPIs are, why they are needed, and how you develop them) and presented the draft protocol for discussion. During the two rounds of the e-Delphi, and the KPI appraisal survey, participants were given the option to answer, ‘not my area of expertise’. The research team, including the Chair of the consensus meetings (DD), also highlighted during each of the meetings that participants should feel that they had received a sufficient explanation about each KPI to inform their voting decisions, that they should feel free to comment or ask questions, or abstain from voting (the latter further to feedback from members during the second consensus meeting). The research team was also conscious, particularly during consensus meetings, that those with lived experience of recurrent miscarriage were engaging in discussions about the merits of various types of investigations and treatments—some for which there was a lack of evidence of benefit—which they may have undertaken, and approached such discussions with sensitivity (e.g. in how discussion framed and evidence presented). Parent advocates/support group representatives were offered a nominal payment, in the form of vouchers, for their role in the RRAG, recognising their contribution to the overall Project. Members were asked to complete a conflict of interest declaration in line with the RE:CURRENT Project Conflict of Interest Policy which documents how interests are declared and conflicts managed and recorded. We did not identify any conflicts that precluded a member from participating in any aspect of the KPI development process.

### Description of the measure development process

**Fig. 1:**
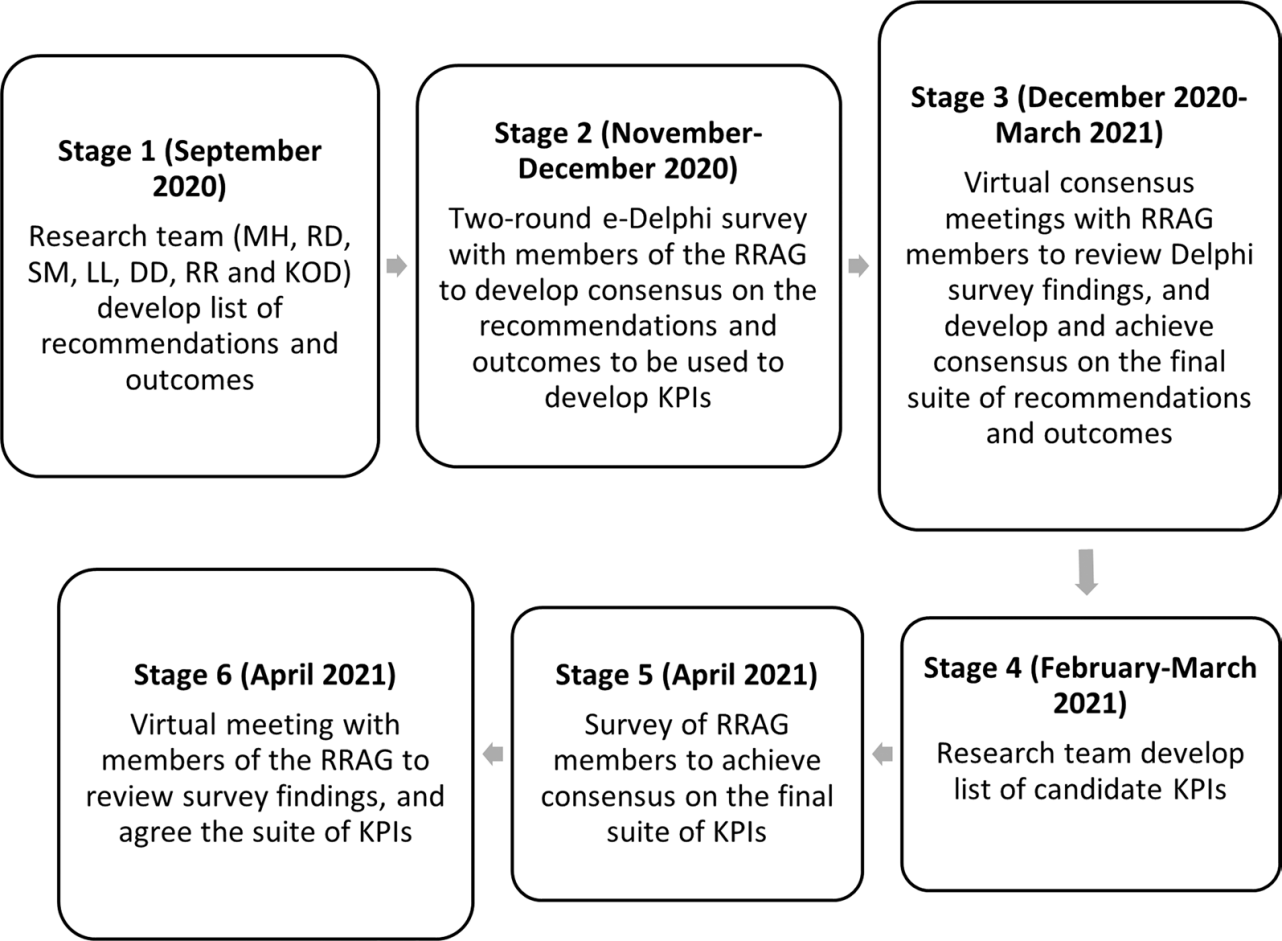
Overview of measure development process and timeline

This consensus activity comprised a six-stage process (see overview in Fig. 1), involving members of the RRAG: (i) identification and synthesis of recommendations for recurrent miscarriage care from a systematic review of clinical practice guidelines in high-income countries [3]; relevant clinical outcomes were identified through the literature and expert opinion (research team members); (ii) two-round modified e-Delphi survey with members of the RRAG to develop consensus on the recommendations and outcomes that should be used to develop KPIs; (iii) four virtual consensus meetings with members of the RRAG to review the findings from the Delphi survey, and develop and achieve consensus on the final suite of recommendations and outcomes that should be included in KPI development; (iv) development of a list of candidate KPIs by the research team; (v) survey of members of the RRAG to achieve consensus on the final suite of KPIs, and (vi) virtual meeting with members of the RRAG to review the survey findings and agree the final suite of KPIs. A similar process has been used to identify and prioritise midwifery care process metrics and indicators [28].

### Stage 1: Development of a list of recommendations and outcomes from which KPIs could be generated

#### Selection of clinical guidelines

We identified clinical practice guidelines for the investigation, management, and/or follow-up of recurrent miscarriage within high-income countries, published between January 2000 and March 2020 (and currently endorsed and/or valid, as specified by the guideline authors and/or professional body) through a systematic review of major databases, guideline repositories, and the websites of professional organisations [3]. The quality of each clinical practice guideline was assessed using the Appraisal of Guidelines for Research and Evaluation (AGREE II) Tool, a validated tool [29]. We did not apply the Guidelines International Network criterion for clinical practice guidelines to determine the eligibility of guidelines for inclusion in our KPI development work [14, 30].

#### Extraction of clinical guideline recommendations

We extracted all recommendations relevant to the investigation, management and follow-up of recurrent miscarriage from included clinical practice guidelines, along with the strength of evidence and/or the grade of recommendation (where available) (data extraction file, available on OSF [31]). We did not place any restriction on including recommendations.

#### Development of a list of recommendations and outcomes

During a half-day meeting, members of the research team (KOD, LL, SM, MH, RD) reviewed and synthesised recommendations from the clinical practice guidelines identified in the systematic review [3], together with findings from qualitative interviews with service providers and women and men with experience of recurrent miscarriage regarding their views and experiences of current services (Dennehy et al., unpublished). No additional items arose in the qualitative findings (i.e. important aspects of RM care that were not already included); therefore, we did not add any further items. We also identified outcome metrics through the extant literature—e.g. outcomes relating to pregnancy loss, gestation and live births identified by Smith and colleagues in their systematic review of outcomes in trials for the prevention and management of miscarriage [32]—and topic/clinical expertise within the research team. This translation process, including decisions taken, was documented by the lead author.

### Stage 2: Development of consensus on the recommendations and outcomes to be included in the development of a final suite of KPIs for recurrent miscarriage care—e-Delphi surveys

The RRAG participated in a multi-phase process involving a two-round e-Delphi survey and a series of consensus meetings to agree on a final list of recommendations and outcomes to be included in developing a final suite of KPIs for recurrent miscarriage care. Delphi survey design facilitates consensus-building on a topic under investigation [33]. An e-Delphi survey involves a series of questionnaires administered electronically in ‘rounds’ to a group of stakeholders to gather their opinions, with results from each round presented to participants in subsequent rounds [28].

We invited members (n = 21) of the RRAG, via email, to participate in Round 1 of the e-Delphi survey (administered via QuestionPro) from 02 to 13 November 2020. The survey contained a brief questionnaire seeking participant data (including name and number of years’ experience relating to recurrent miscarriage) and a list of recommendations and outcomes, divided into five categories: structure of care, counselling/supportive care, investigations, treatment, and outcomes. Each recommendation and outcome was accompanied by the quality of evidence, where available, and the number of clinical practice guidelines which contained the particular recommendation. Participants were also invited to add any further recommendations and/or outcomes they considered important or relevant for inclusion. We asked participants to rate the extent to which the realisation of a recommendation or outcome was important for measuring quality care for recurrent miscarriage, using a nine-point Likert scale (1–3 = not important, 4–6 = unsure of importance and 7–9 = important); similar wording was used by van den Berg and colleagues when developing guideline-based KPIs for early pregnancy assessment units [19]. Participants were also invited to list their top five recommendations or outcomes for each category, in order of importance from 1 to 5 (see Additional file 2 for a sample survey item). We provided participants with a PDF copy of the survey, which contained a glossary of key terms (e.g. Delphi study, KPI, and outcome), advising that it might be helpful to read in advance of completing the online survey. Consensus on the inclusion of a recommendation or outcome was determined where 70% or more participants rated the recommendation or outcome as 7–9 and less than 15% of participants rated the recommendation or outcome as 1–3; this criteria is used for developing core outcome sets in healthcare (http://www.comet-initiative.org/). Results from Round 1 were collated and used to inform the development of the second round of the e-Delphi survey.

In the second round, administered via QuestionPro from 23 November to 04 December 2020, we presented all participants with the same recommendations and outcomes as those presented in Round 1. In this round, however, we included how the Group rated each recommendation and outcome during Round 1, presenting the overall rating results (percentages) for each; see Additional file 3 for a sample survey item. In the invitation email to each participant, we also provided a separate file containing confidential details of their own ratings for each item. We asked them to consider these ratings and then to rate the importance of each recommendation and outcome again, revising their rating based on how others had rated them, if they wished. As in Round 1, consensus on inclusion of a recommendation or outcome was determined where 70% or more participants rated the recommendation or outcome as 7–9 and less than 15% of participants rated the recommendation or outcome as 1–3. Participants were advised that each survey would take approximately 45–60 min to complete.

### Stage 3: Development of consensus on the recommendations/outcomes to be included in the development of a final suite of KPIs for recurrent miscarriage care—consensus meetings

A series of four, three-hour, virtual consensus meetings (09 December 2020; 12 January, 03 February, 03 March 2021) were held with members of the RRAG to discuss the e-Delphi survey findings and develop and achieve consensus on the final suite of recommendations and outcomes that should be included in KPI development. Meetings were chaired by DD, an experienced facilitator of such meetings. We presented participants with the percentage rating for each recommendation and outcome, overall and by stakeholder group from both rounds of the e-Delphi survey via PowerPoint presentation (see Additional file 4 for an example). We did not exclude any recommendations or outcomes based on the ratings received during the e-Delphi survey as we wanted to give participants the opportunity to review and discuss each of them as a group (including the overall ratings, and ratings by stakeholder group), and to ask any questions and re-evaluate any items, prior to excluding any from further consideration. Further to discussion and agreement within the Group, KOD provided a lay explanation for each recommendation and outcome where members felt that greater explanations were needed prior to them making an informed vote. Participants then discussed their views, before voting on whether they felt that each should be included in the final suite of recommendations and outcomes. To be retained, a recommendation or outcome required a yes vote by ≥ 70% of participants. Detailed notes of each meeting, including decisions taken and voting results, were taken by MH and verified by the team.

### Stage 4: Translation of recommendations into candidate KPIs

Members of the research team (MH, LL and KOD) reviewed the reports from the consensus meetings before translating the agreed key recommendations and outcomes into candidate KPIs; these were then reviewed by remaining members of the research team (RD, DD, RR and SM). For each KPI, we generated the following details: title; number; description; rationale; calculation, comprising a numerator divided by a denominator expressed as a percentage. We detailed the numerator and denominator for each KPI, outlining any exceptions (e.g., age, contraindications) where applicable. Outcome-related KPIs were framed as outcomes which a recurrent miscarriage clinic/service should report/audit. Outcomes related to complications in future pregnancies (e.g. preterm birth, fetal growth restriction, and stillbirth) were included, given that recurrent miscarriage is an important indicator for such complications [1], not that they indicate quality of recurrent miscarriage care per se. We considered the potential for KPIs to be integrated into existing coding and data systems; however, participants highlighted that the data collection systems within the Irish health system were not sufficiently developed in this regard. This translation process, including steps and decisions taken, were documented by MH.

The KPIs will be used to evaluate recurrent miscarriage services in all 19 maternity units/hospitals across the Republic of Ireland as part of the RE:CURRENT study, and were formulated as such. While they are being developed for research purposes to understand what care is currently provided to those who experience recurrent miscarriage, the KPIs may also be used to inform quality improvement efforts at local, regional and national levels; they will not be used for accountability purposes (e.g. pay-for-performance).

### Stage 5: Achieving consensus on the final suite of KPIs—survey

We invited 20 members of the RRAG, via email, to participate in a final survey from 14 to 26 April 2021, to appraise each indicator and reach a consensus on the final suite of KPIs. The email included a link to a 10-min video outlining the purpose of the survey and what they were being asked to do, along with a Word version of the survey—given its length, the lack of a ‘save and continue later’ facility within the free version of QuestionPro, and feedback from participants from the previous e-Delphi rounds—to be completed and returned via email.

The survey contained details of the KPIs developed in Stage 4, by category. At the end of each category/section, we noted details of recommendations or outcomes that were rated as important to include in KPI development by the RRAG in Stage 3, but which were not translated into a KPI, with reasons noted. For example, if there was another similar recommendation, or the recommendation did not translate well into a KPI, i.e. it could not tangibly be measured by a KPI.

#### Measure appraisal

We asked participants to review each KPI using a framework to help them judge if a KPI was appropriate for inclusion in the final suite of KPIs (see Table 1). We asked them to select YES, NO, or DON’T KNOW, for each of four criteria on which the KPI was being assessed: process-based, important, operational, feasible. Based on their assessments, we then asked them to vote on whether they felt that the KPI should be included in the final suite. As with preceding stages, participants were given the option to exempt themselves from rating a particular KPI/criterion if they felt it was outside of their area of expertise. (See Additional file 5 for a sample survey item). Consensus on the inclusion of a KPI was determined where 70% or more participants rated the KPI for inclusion in the final suite.

**Table 1:** QCM judgement framework tool^a^

Domain	Description
Process-focused	The metrics/indicator contributes clearly to the measurement of recurrent miscarriage care processes
Important	The data generated by the metric/indicator will likely make an important contribution to improving recurrent miscarriage care processes
Operational	The indicator is quantifiable (i.e. can be measured); definitions are precise, and reference standards are developed and tested or it is feasible to do so
Feasible	It is feasible to collect and report data for the metric/indicator in the relevant setting

^a^Adapted from Flenady and colleagues [34] and Devane and colleagues [28]

### Stage 6: Virtual meeting to review the survey findings and agree the final suite of KPIs

At a further virtual meeting, the KPI appraisal survey results were presented to and discussed with RRAG members, and the final suite of KPIs agreed. As with other meetings, all RRAG members received emails with supporting documentation before and after this final meeting, and had the opportunity to review the final list and contribute to discussions. At this meeting, feedback was also sought on the KPI development process. Members present were invited to think of three words that came to mind when they thought about the process and to enter these into Mentimeter (interactive virtual presentation app; https://mentimeter.com); a word cloud was generated and discussed. Participants were then asked to consider what worked well and what could be done better, and to write their thoughts (anonymously) on two virtual bulletin boards on Padlet (https://padlet.com). SM summarised key themes arising and invited discussion. RRAG members were invited to add any further thoughts to the Padlet walls for a week after the meeting to enable those unable to attend the meetings to contribute their feedback.

Finally, quantitative and qualitative data generated during each stage were entered into Microsoft Excel and verified. Quantitative data were summarised descriptively, while qualitative data was analysed thematically [35].

## Results

### Participation during various stages of KPI development

Twenty-one members of the RRAG took part, to varying extents, in Stages 2 and 3 of the consensus-building activities. Ninety-five percent of members (20/21) took part in Round 1 of the e-Delphi survey, while 90% (19/21) took part in Round 2 (Stage 2). The bold designates the total numbers for each row above—emboldened to make it stand out. Round 1 had 273 years’ experience (clinical/lived) related to recurrent miscarriage (Mean = 13.7 years; Range = 5–25 years), compared with 265 years’ experience (Mean = 13.9 years; Range = 5–25 years) in Round 2. All available members were invited to take part (i.e. had voting rights) in the consensus meetings (Stage 3), along with two members of the research team (KOD and LL). Participation in these meetings varied, with 48%, 78%, 70% and 70% participating in meetings 1 to 4, respectively (Table 2). Participation by stakeholder group varied across the four meetings, as follows: health professionals (38–85%); management/governance role (33–50%); parent advocate/support group representatives (75–100%). Fourteen out of 20 RRAG members (70%) completed the final KPI appraisal survey (Stage 5). In contrast, ten (50%) participated in the final meeting to review the survey findings and agree on the final suite of KPIs (Stage 6).

**Table 2:** Overview of participants in the KPI development process

Participant group	Total	Delphi round 1		Delphi round 2		Total	Consensus meeting 1		Consensus meeting 2		Consensus meeting 3		Consensus meeting 4		Total	Final survey		Final review meeting	
		n	%	n	%		n	%	n	%	n	%	n	%		n	%	n	%
Health professional	11	10	91	9	82	13^a^	5	38	11	85	11	85	10	77	10	7	70	5	50
Management/governance role	6	6	100	6	100	6	3	50	3	50	2	33	3	50	6	4	67	2	33
Parent advocate/support group representative	4	4	100	4	100	4	3	75	4	100	3	75	3	75	4	3	75	3	75
**Total**	**21**	**20**	**95**	**19**	**90**	**23**	**11**	**48**	**18**	**78**	**16**	**70**	**16**	**70**	**20**	**14**	**70**	**10**	**50**

The bold designates the total numbers for each row above

^a^Two of the study investigators (KOD and LL) participated in voting during the consensus meetings, given their clinical and research expertise in recurrent miscarriage

### KPI development

**Fig. 2:**
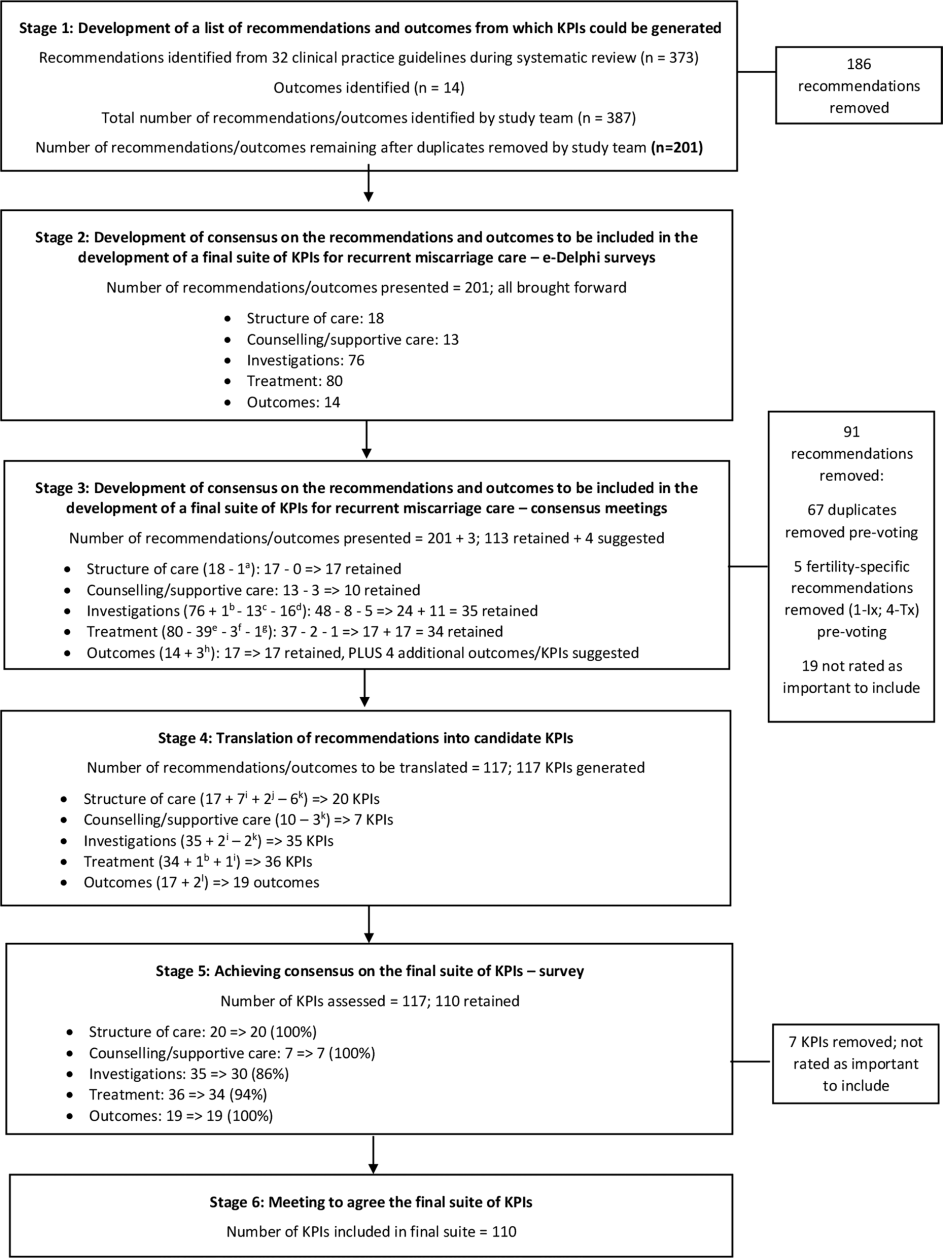
Stages in the development of guideline-based KPIs for recurrent miscarriage care. *Notes*: ^a^Moved to Investigations category, ^b^Moved from Structure of care category, ^c^Removed during consensus meeting 2 due to overlap with one or more other recommendations, ^d^Removed by research team members (KOD + MH) before consensus meeting 3 due to overlap with one or more other recommendations, ^e^Removed by research team members (KOD + MH) before consensus meeting 3 due to overlap with one or more other recommendations, ^f^Removed during consensus meeting 3 due to overlap with one or more other recommendations, ^g^Removed during consensus meeting 4 due to overlap with one or more other recommendations, ^h^One outcome was divided into four outcomes during consensus meeting 4, ^i^More than one KPI generated for some recommendations, ^j^KPIs developed for items suggested by RRAG moved from Outcomes into this section, ^k^Recommendation/outcome not translated into a KPI (duplicate/covered somewhat by another KPI; did not translate well into a KPI), ^l^KPI generated for additional items suggested by RRAG

Each stage of the consensus process, including numbers of removed/reduced included/excluded during each, is outlined in Fig. 2.

### Stage 1: Development of a list of recommendations and outcomes from which KPIs could be generated

We identified 373 recommendations from the 32 clinical practice guidelines included in our systematic review and 14 potentially relevant outcomes from the extant literature and expertise within the study team, giving a total of 387 (Fig. 2). Members of the study team agreed a final list of 201 recommendations and outcomes.

### Stage 2: Development of consensus on the recommendations and outcomes to be included in the development of a final suite of KPIs for recurrent miscarriage care—e-Delphi surveys

Twenty members of the RRAG rated the importance of the 201 recommendations and outcomes in Round 1 of the e-Delphi survey, while 19 of them re-rated these again in Round 2 (Table 2). While participants were advised that surveys would take 45–60 min to complete, some reported that it took much longer. Levels of consensus on the importance of recommendations and outcomes for inclusion increased across both rounds, except for ‘structure of care’ as all items there were rated as important in both rounds (Table 3).

**Table 3:** Number of recommendations/outcomes voted as important to include (i.e. ≥ 70% voted yes to inclusion) after each round of the e-Delphi survey

Category	Round 1		Round 2	
	No. voted as important/Total no.	%	No. voted as important/Total no.	%
Structure of care	18/18	100	18/18	100
Counselling/supportive care	8/13	62	9/13	69
Investigations	26/76	34	33/76	43
Treatment	17/80	21	37/80	46
Outcomes	7/14	50	10/14	71

In general, participants voted on most, if not all, recommendations within ‘structure of care’, ‘counselling/supportive care’ and ‘outcomes. For ‘investigations’ and ‘treatment’, health professionals voted on more items (Additional file 6). For example, the mean number of recommendations voted on by health professionals in the ‘treatment’ category (n = 80) in Round 2 was 67.1 (range = 11–80), compared with 20.3 for those in management/governance (range = 0–64), and 28 for parent advocates/support group representatives (range = 0–60).

Participants suggested additional recommendations and/or outcomes for inclusion during each round (R), relating to structure of care (R1: 11; R2:7), counselling/supportive care (R1: 4; R2: 1), treatment (R1: 1), and outcomes (R1: 1; R2: 2). We did not add any additional recommendations or outcomes as the suggestions were either already covered within other items, or they were more suited to being included in a clinical practice guideline.

We did not analyse the ranking data (i.e. top five recommendations and outcomes for each category) provided by participants, primarily because many participants did not complete this activity, stating that it was outside their expertise.

### Stage 3: Development of consensus on the recommendations and outcomes to be included in the development of a final suite of KPIs for recurrent miscarriage care—consensus meetings

All 201 recommendations and outcomes presented in the two rounds of the e-Delphi survey were brought forward to the consensus meetings. Throughout these four meetings, through discussion and voting, this number was reduced to 113, across the five categories: structure of care [17]; counselling/supportive care [10]; investigations [35]; treatment [34]; outcomes [17], and 4 additional outcomes/KPIs were suggested (Fig. 2).

### Stage 4: Translation of recommendations into candidate KPIs

The research team generated 117 KPIs from the 113 recommendations and outcomes agreed during Stage 3 and the four additional outcomes/KPIs suggested (Fig. 2). Some of the recommendations and outcomes were not translated into KPIs as they were duplicates or covered by another KPI or did not translate well into a KPI.

### Stage 5: Achieving consensus on the final suite of KPIs—survey

Fourteen out of twenty RRAG members completed the final survey (Table 2), which resulted in 110 of the 117 KPIs being retained (Fig. 2; details of KPIs retained and excluded in Table 4 and Additional file 7, respectively). Additional comments noted by participants included concerns about putting some recommendations into practice, i.e. feasibility of recommendations rather than measuring a particular care process (e.g. clinic location, format/language of information; standardised templates); the feasibility of measuring certain KPIs, especially those with multiple components; the framing of some ‘negative’ KPIs, i.e. instances where a particular investigation or treatment should not be undertaken; the ability of clinics to conduct follow-ups to collect outcome data. There were also suggestions for merging KPIs, e.g. referral criteria & specification, investigation & management plans. These were discussed in Stage 6; no amendments were made, and some suggestions were noted for incorporation into later guideline development.

**Table 4:** Agreed list of KPIs for recurrent miscarriage care

KPI No.	KPI title	KPI sub-category
KPI category: Structure of care (n = 20)		
1.4(a)	Dedicated recurrent miscarriage clinic on-site	Dedicated clinic
1.4(b)	Access to dedicated recurrent miscarriage clinic	Dedicated clinic
1.6(a)	Core recurrent miscarriage team	Staffing/expertise
1.6(b)	Access to psychological supports	Staffing/expertise
1.12(b)	Staff education and training	Staffing/expertise
1.12(a)	Care experience	Staffing/expertise
1.5	Location of dedicated recurrent miscarriage/pregnancy loss/gynaecology clinic	Location/equipment/facilities
1.10	Laboratory services	Location/equipment/facilities
1.3	Formal referral process	Referral structures
1.2(a)	Referral criteria	Referral structures
1.2(b)	Specification of referral criteria	Referral structures
1.18	Education/information for health professionals about referral processes	Referral structures
1.001	Referral sources	Referral structures
1.002	Referral times	Referral structures
1.1	Timing of investigations	Referral structures
1.14(a)	First visit—written information about what to expect	Information provision and plans
1.14(b)	Written information about sources of support	Information provision and plans
1.14(c)	Written information about recurrent miscarriage	Information provision and plans
1.17(a)	Tailored investigation plan	Information provision and plans
1.17(b)	Tailored management plan	Information provision and plans
KPI category: Counselling/supportive care (n = 7)		
2.9	Information provision—risk factors: advancing age	Information provision
2.10	Information provision—modifiable risk factors	Information provision
2.5	Unexplained recurrent miscarriage—information about prognosis	Information provision
2.11	Information provision—unorthodox investigations/treatments	Information provision
2.7	Information provision—treatment uncertainty	Information provision
2.13	Clinical trials	Information provision
2.12	Genetic counselling	Genetic counselling
KPI category: Investigations (n = 30)		
3.1	Medical, obstetric and family history	Standard investigations
3.2	Information about behavioural and weight-related risk factors	Standard investigations
3.15	Full blood count	Standard investigations
3.16	Electrolytes and liver function tests	Standard investigations
3.4	Assessment of uterine anatomy	Anatomical investigations
3.7	Assessment of uterine anatomy using transvaginal ultrasound	Anatomical investigations
1.9	Access to 3D ultrasound	Anatomical investigations
3.12	Imaging/imaging with hysteroscopy to diagnose uterine septa	Anatomical investigations
3.14	Complete investigation following Müllerian uterine malformation diagnosis	Anatomical investigations
3.20	Measurement of antinuclear antibodies testing	Immunological screening
3.21	Natural killer cell testing	Immunological screening
3.27(a)	Routine screening for hereditary thrombophilia	Haematology
3.27(b)	Screening for hereditary thrombophilia, with risk factors	Haematology
3.34(a)	Screening for antiphospholipid syndrome—routine	Haematology
3.34(b)	Screening for antiphospholipid syndrome—non-routine	Haematology
3.36	Monitoring of plasma coagulation markers	Haematology
3.25	Thyroid stimulating hormone, thyroid peroxidase-antibodies, and thyroxine (T4) testing	Metabolic & endocrinologic factors
3.38	Screening for diabetes	Metabolic & endocrinologic factors
3.45	Ovarian reserve testing	Metabolic & endocrinologic factors
3.48	‘Day 2–5’ hormone profile	Metabolic & endocrinologic factors
3.46	Luteal phase insufficiency testing	Metabolic & endocrinologic factors
3.47	Androgen testing	Metabolic & endocrinologic factors
3.55	Vitamin D measurement	Metabolic & endocrinologic factors
3.51	Infectious screening	Infectious screening
3.60	Cytogenetic analysis of pregnancy tissue at the third miscarriage	Screening for genetic factors
3.58	Array-based comparative genomic hybridisation (Array-CGH)	Screening for genetic factors
3.57	Genetic polymorphism study	Screening for genetic factors
3.64	Peripheral karyotyping	Screening for genetic factors
3.65	Cytogenetic testing of both parents	Screening for genetic factors
3.71	Testing for spermploidy/DNA fragmentation	Screening for male factors
KPI category: Treatment (n = 34)		
4.2a	Treatment of antiphospholipid syndrome—referral to local haematology service	Antiphospholipid syndrome
4.2b	Treatment of antiphospholipid syndrome—low dose aspirin and heparin in next pregnancy	Antiphospholipid syndrome
4.4	Treatment of antiphospholipid syndrome—intravenous immunoglobulin therapy	Antiphospholipid syndrome
4.25	Treatment of subclinical hypothyroidism with levothyroxine	Recurrent miscarriage with metabolic and endocrinologic factors
4.28	Treatment of overt hypothyroidism with levothyroxine	Recurrent miscarriage with metabolic and endocrinologic factors
4.30	Treatment of women with subclinical hypothyroidism in next pregnancy	Recurrent miscarriage with metabolic and endocrinologic factors
4.31	Treatment of women with thyroid autoimmunity and hypothyroidism in next pregnancy	Recurrent miscarriage with metabolic and endocrinologic factors
4.40	Human chorionic gonadotrophin supplementation in pregnancy	Recurrent miscarriage with metabolic and endocrinologic factors
4.36	Bromocriptine treatment in women with recurrent miscarriage and hyperprolactinemia	Recurrent miscarriage with metabolic and endocrinologic factors
4.42	Metformin supplementation	Recurrent miscarriage with metabolic and endocrinologic factors
4.14	Preimplantation genetic testing	Recurrent miscarriage with genetic background
4.12	Unexplained recurrent miscarriage: Preimplantation genetic screening with in vitro fertilisation treatment	Recurrent miscarriage with genetic background
4.8	Oocyte donation	Recurrent miscarriage with poor ovarian reserve
4.23	Sperm selection	Recurrent miscarriage with male factor
4.67	Myomectomy (laparoscopic or open)	Uterine factors in recurrent miscarriage
4.68	Hysteroscopic septum resection	Uterine factors in recurrent miscarriage
4.71	Metroplasty in women with bicorporeal uterus and double cervix	Uterine factors in recurrent miscarriage
4.70	Uterine reconstruction for hemi-uterus	Uterine factors in recurrent miscarriage
1.7	MDT for hysteroscopic metroplasty of a uterine septum	Uterine factors in recurrent miscarriage
4.74	Surgical removal of intrauterine adhesions	Uterine factors in recurrent miscarriage
4.43	Use of antibiotics	Antibiotics
4.61	Unexplained recurrent miscarriage: Supportive care	Unexplained recurrent miscarriage
4.54	Unexplained recurrent miscarriage: Use of low molecular weight heparin or low dose aspirin	Unexplained recurrent miscarriage
4.1	Corticosteroids (e.g. prednisolone)	Unexplained recurrent miscarriage
4.19	Glucocorticoids (clinical studies)	Unexplained recurrent miscarriage
4.52	Intravenous immunoglobulin	Unexplained recurrent miscarriage
4.27	Empiric progestogen	Unexplained recurrent miscarriage
4.57	Unexplained recurrent miscarriage: Intralipid therapy	Unexplained recurrent miscarriage
4.51	Unexplained recurrent miscarriage: Lymphocyte immunisation therapy	Unexplained recurrent miscarriage
4.58	Unexplained recurrent miscarriage: Granulocyte colony-stimulating factor	Unexplained recurrent miscarriage
4.17	Unexplained recurrent miscarriage: Immunotherapy	Unexplained recurrent miscarriage
4.22	Unexplained recurrent miscarriage: Therapy with tumour necrosis factor-α receptor blockers	Unexplained recurrent miscarriage
4.59	Unexplained recurrent miscarriage: Endometrial scratching	Unexplained recurrent miscarriage
4.55	Unexplained recurrent miscarriage: Folic acid	Unexplained recurrent miscarriage
KPI category: Outcomes (n = 19)		
5.1	New pregnancy	New pregnancy
5.2a	New pregnancy: Spontaneous conception	New pregnancy
5.2bi	New pregnancy: Ovulation induction and intrauterine insemination	New pregnancy
5.2bii	New pregnancy: IVF and IntraCytoplasmic Sperm Injection	New pregnancy
5.2biii	New pregnancy: IVF and IntraCytoplasmic Sperm Injection with donor gametes	New pregnancy
5.2biv	New pregnancy: Any use of Preimplantation Genetic Testing	New pregnancy
5.3	New pregnancy reaches 2^nd^ trimester	New pregnancy
5.4	New pregnancy reaches 3^rd^ trimester	New pregnancy
5.5	New pregnancy: Avoidance of fetal growth restriction	New pregnancy
5.6	New pregnancy: Avoidance of placental abruption	New pregnancy
5.7	New pregnancy: Avoidance of pre-eclampsia	New pregnancy
5.8	New pregnancy: Avoidance of preterm birth	New pregnancy
5.9	New pregnancy: Avoidance of stillbirth	New pregnancy
5.10	New pregnancy: Avoidance of neonatal death	New pregnancy
5.11	New pregnancy: Treated as high risk, with consultant-led care	New pregnancy
5.12	Interval to next pregnancy: < 6 months	New pregnancy
5.13a	Interval to next pregnancy: ≥ 6 months and < 12 months	New pregnancy
5.13b	Interval to next pregnancy: < 12 months	New pregnancy
5.16	New pregnancy: Attend early pregnancy clinic/have an early pregnancy scan	New pregnancy

As with the recommendations and outcomes, participants generally voted on most, if not all, recommendations within ‘structure of care’, ‘counselling/supportive care’ and ‘outcomes’ (except those in management/governance roles for the latter). Similarly, for ‘investigations’ and ‘treatment’, health professionals voted on a greater number of items but were followed closely by parent advocates/support group representatives on this occasion (Additional file 8).

### Stage 6: Virtual meeting to review the survey findings and agree on the final suite of KPIs

Ten RRAG members attended the final meeting and approved the final list of 110 KPIs, agreed during Stage 5 (Table 4).

### Feedback from participants on the KPI development process

**Fig. 3:**
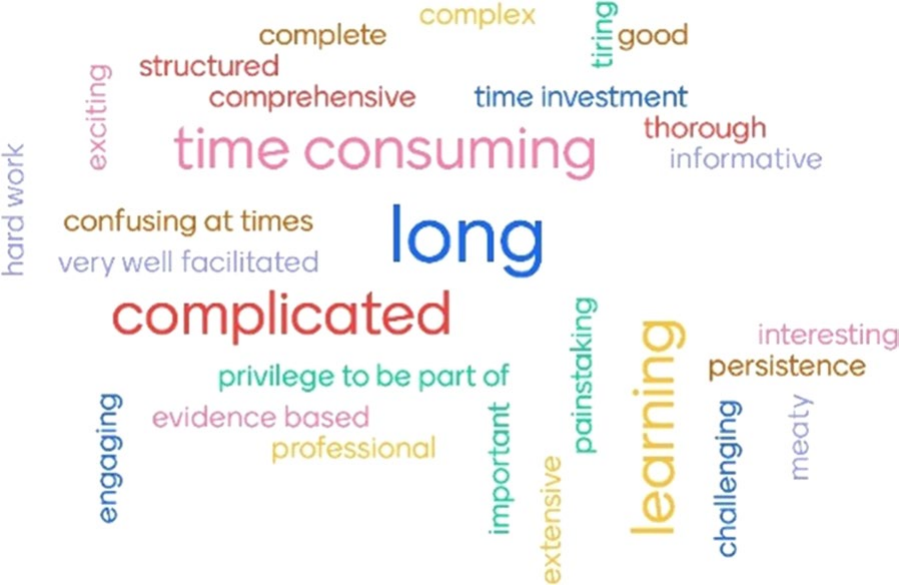
Word cloud: words that come to mind when thinking about the KPI development process

The word cloud generated from participants’ feedback on the KPI development process is presented in Fig. 3. Words that predominated related to the long, complicated, time-consuming process; despite this, positives were noted relating to comprehensiveness, good facilitation, learning, and engagement/participation.

These findings were further elaborated on in participants’ responses to the questions posed around what worked well and what could be done differently; participant quotes are designated by identifiers W(ell) and B(etter), relating to the latter. We generated three themes: accessibility, richness in diversity, streamlining the development process.

*Richness in diversity* describes the benefits stated by some participants, including multiple/diverse perspectives, the rich discussions, learning (which they may have missed out on if they knew the time commitment involved initially; they would have ‘baulked at the outset’), and how it ‘gives the project a lot of weight’.

“The discussions which flowed during the meetings were brilliant, and the knowledge and passion of the people on the group is inspirational. I am delighted to be part of the group” (W3)

Within this theme, some areas for improvement were noted, such as the benefit of having more representation from doctors/midwives in training and more

“alternative voices to the medical expertise that was on the group but were equally informed in terms of fertility, miscarriage”

(B7).

*Accessibility* represents the majority of comments received and describes what facilitated participants to access/engage with the process, or not. Sub-themes encompassed: skilled facilitation, communication with/from the research team, virtual access/timing of meetings, and making the process more user-friendly. Participants valued the skilled facilitation during the consensus meetings, lay explanations provided, and adequate time for discussion.

“Some of the consensus meetings were….......heavy and I sometimes felt I was overwhelmed with all the medical jargon, but Keelin's [KOD’s] explanations were super as well as Declan's [DD’s] and other members in the group” (W5)

Some thought that providing a reference guide with an explanation of medical terms at the outset would have been very beneficial. One parent advocate felt that

“the responsibility felt heavy at times, to raise questions from a parent perspective without the medical expertise”

and suggested that it would have been helpful for the parent representatives to have met together with the team, prior to starting the process to consider their role in it and to strengthen their voice perhaps (B13). A few participants stated that the time commitment involved should have been made clearer at the outset.

Participants highlighted positives regarding the responsiveness/accessibility of research team members (email/phone communication) when information/clarification was needed, updates regarding progress and information in advance, and honesty around the challenges experienced during the development process. Many felt that the virtual format, and evening meetings, facilitated access; the shorter (3-h) meetings, rather than one long day, were generally preferred. The sub-theme ‘making the process more user-friendly’ related to comments from a few participants about the difficultly experienced with the Delphi survey, including the inability to ‘save and continue later’ on the online platform, as well as one noting that the ability to abstain from voting during consensus meeting should have been clearer at the outset.

*Streamlining the development process* captures comments made by a few participants about how the number of recommendations/KPIs could have been narrowed down—by those with the relevant expertise—before asking all participants to vote on them.

“Perhaps the KPIs could have been narrowed down by those who really had the expertise to do that prior to the big group coming together to vote on them - or else, depending on people's backgrounds, being invited to come for voting on sections that were only within people's expertise/experience.” (B16)

## Discussion

Standardised care pathways tailored to women/couples who experience recurrent miscarriage are needed to improve care; however, clinical practice is inconsistent and poorly organised. In this paper, we outline how we developed guideline-based KPIs for recurrent miscarriage care, following established guidance [14], to be used to evaluate recurrent miscarriage services in Ireland. Through a six-phase consensus-building process, we developed 110 KPIs, which are well distributed across the five categories, including structures and outcomes, in addition to investigations and treatments. Indicators were developed with the RRAG, a multi-stakeholder group comprising health professionals, those involved in the administration, governance and management of maternity services, and parent advocates/support group representatives. To date, such exercises have generally only involved clinicians, with the need for greater stakeholder involvement highlighted [13, 20]. We sought to establish the feasibility of developing guideline-based indicators with a diverse stakeholder group, including those with lived experience of recurrent miscarriage, and share insights into our collective experiences. We will now explore these further, guided by themes generated from participants’ insights.

### Richness in diversity

Guideline-based KPI development is often only conducted with clinicians [18, 19], with lack of patient involvement a noted limitation [13, 20], even within guideline development itself [3]. We found that it is possible to involve a range of stakeholders in developing such indicators, including women and men with lived experience of recurrent miscarriage. Participants in our study valued the opportunity to be involved and to discuss and learn from each other, being afforded the ‘space to talk’ [36], though perhaps some contributors did not feel that they brought equally important knowledge as others. As noted by Rushforth and colleagues [17], we found that, following lay explanations and rich discussions during the consensus meetings, parent advocates/support group representatives could rate complex recommendations/KPIs and often gave similar opinions as health professionals.

### Accessibility

The above considered, participant feedback nonetheless suggested that the clinical voice was perhaps strongest throughout; whether this influenced voting during the consensus meetings is unknown. Indeed, Williams and colleagues argue that ‘professional advantage’ will always be held in patient and public involvement despite efforts to counter such impacts and a willingness to share power [37]. The latter is often incompatible with the traditional research structures in which involvement takes place [22, 38, 39], as well as what knowledge is deemed as legitimate [22, 38]. It should also be noted that there were variations within professional groupings also; with non-clinicians (whether part of the ‘health professional’ or ‘management/governance’ groups) abstaining from voting to similar extents to parent advocates/support group representatives during both rounds of the Delphi survey.

Given the initial volume of recommendations and outcomes, we held discussions with the collective group of stakeholders, and there was benefit in having diverse perspectives shared. Smaller group discussions, by stakeholder group—particularly those with lived experience—would have been useful in advance of larger group discussions, as noted by participants. This would have increased the time commitment and should be factored into planning, and resourcing, similar projects. The need to be realistic and upfront about the time commitment for such activities at the outset, was highlighted by participants during feedback. This was an ongoing challenge for the research team throughout, balancing the (sometimes unanticipated) volume of work, the need and desire for meaningful involvement, and participants’ time. This was particularly evident when synthesising the recommendations during Stage 1; many were retained as the team did not wish to influence how participants perceived/voted on them—these were further synthesised during Stage 3. It would perhaps be useful to have a meeting at the outset with participants to discuss how such issues would be handled, particularly regarding areas such as recurrent miscarriage where there is a lack of consensus [3, 40]. This could include how participants could be most meaningfully involved [22], how recommendations would be selected, and providing lay explanations—or how such explanations could be facilitated (including perhaps an independent provider of same). This may also enable participants to engage more actively and/or meaningfully in voting during Delphi survey rounds.

Despite the challenging, lengthy process, conducted during the COVID-19 pandemic, there was a relatively high participation rate amongst the diverse stakeholder group throughout (approximately 70% for many of the stages), despite a decline during the latter stages, particularly amongst those in management and governance roles. The high level of commitment and involvement of participants was facilitated, certainly in part, by the skilled facilitation including lay explanations and time for discussion, open communication with/from research team, and the virtual access and timing of meetings. Virtual meetings may also have reduced power differentials between the research team and participants as we shared insights into our personal spaces/lives during calls [41]. However, it is also possible that virtual meetings limited engagement with some contributors [42].

### Streamlining the development process

As noted by other researchers, developing KPIs can be time consuming and resource-intensive [16, 17, 43], as was the case here. We started the process with many recommendations (n = 373) and outcomes (n = 14), in the absence of a national guideline for recurrent miscarriage. Participants had to agree recommendations for recurrent miscarriage care, before consensus on which recommendations should be prioritised for KPI development.

Furthermore, the research team did not exclude any guidelines/recommendations based on quality assessment/AGREE II scores. Ideally, indicator development should be conducted as part of the guideline development process and originate from evidence-based guideline recommendations [44]. One hundred and ten KPIs was a large number of indicators to include in the final suite, though similar numbers have been generated for midwifery care processes [28]. Similar to Fiset and colleagues [43], some of the included guidelines focused on specific aspects of recurrent miscarriage care; others were broader in focus, which may be more helpful in guiding practice and identifying KPIs. In developing KPIs for recurrent miscarriage care, van den Boogaard and colleagues noted that degree of acceptance of an indicator diminished with a decrease in evidence level; however some ‘authority-based statements’ were selected as indicators, potentially because they are part of everyday clinical practice [18]. In our systematic review, we identified much variation in grading systems used to rate the quality of evidence within each of the included guidelines and how the levels of evidence were presented in each, with inconsistency in levels of evidence and strength of recommendations across similar recommendations [3], also noted by Fiset and colleagues [43]. The KPIs generated in our study, were developed from recommendations with varying levels of evidence. Those which recommended against particular practices were particularly challenging for participants to engage with; however the importance of including these was agreed during consensus meetings, given that women/couples with recurrent miscarriage can undergo unnecessary investigations and/or treatments [2].

### Strengths and limitations

We used a systematic, pre-determined, approach to generate KPIs for recurrent miscarriage care. While these were developed for the Irish context, our findings have broader relevance internationally. The inclusion of a diverse stakeholder group, particularly parent advocates/support group representatives, and the focus on processes are key strengths of this study. Such diversity in development panels is advocated; it includes those with an interest in the results of the study, in addition to the potential for diverse perspectives about quality of care which can enrich the results [45]. We had a relatively high participation rate amongst the diverse stakeholder group throughout which is also a strength of this study, as discussed in more detail above.

Several potential limitations should be noted. We did not apply any criteria to select recommendations from the guidelines (e.g. level of evidence or strength of the recommendation). Not all quality indicator development criteria may be addressed in guideline development [44]. Nothacker and colleagues recommend consideration of relevance (as a minimum: potential for improvement/clinical relevance), scientific soundness (as a minimum: the evidence supporting the measure), and feasibility (as a minimum: clarity of definition and measurability) when deciding on the most appropriate quality indicators [14]. In this case, relevance and feasibility played a central role, more so than scientific soundness, given the low evidence supporting many of the included guideline recommendations [43]; however, levels of evidence were considered by participants during all stages of development. While we had a high level of participation, and good representation, during each stage, all members of the RRAG could not attend all meetings and therefore were unable to participate in some discussions which may have influenced their interpretation and rating of some of the recommendations, outcomes and/or KPIs. All members did receive emails and supporting documentation before and after meetings however, and had opportunity to contribute to discussions. Finally, our KPIs were developed based on a systematic review of clinical guidelines for recurrent miscarriage within high income countries, given the large discrepancies in pregnancy outcomes and care structures between high, low and middle-income countries [46, 47] and variation in country-specific models of recurrent miscarriage according to healthcare system structures and resources [2]. Further work would be needed to assess the suitability of the KPIs for such contexts, including any potential adaptations, and subsequent measure appraisal. Insights into our processes and experiences of developing guideline-based KPIs for recurrent miscarriage care with a diverse stakeholder group certainly have global relevance and could be use within low and middle income countries to guide similar efforts.

### Deviations from protocol

The main deviations from the original protocol included: (i) additional consensus meetings, given the volume of recommendations extracted and the extra time required for discussion and voting for consensus-building, and (ii) an extra stage, encompassing a final survey to before—instead of during—the final meeting to review, develop and achieve consensus on the final suite of KPIs given the volume of indicators to be assessed.

## Conclusions

From an initial list of 373 recommendations and 14 outcomes, 110 KPIs across the following five categories, were prioritised for inclusion in a suite of guideline-based KPIs for recurrent miscarriage care: (i) structure of care (n = 20); (ii) counselling and supportive care (n = 7); (iii) investigations (n = 30); treatment (n = 34); outcomes (n = 19). The identified KPIs will now be used to assess the quality of recurrent miscarriage care provided in all 19 maternity hospitals/units in the Republic of Ireland; they will be pilot tested at one site prior to administration across all sites. Data and KPIs generated through the various stages will also contribute to the development of a national clinical practice guideline for recurrent miscarriage care. It is important, and feasible, to develop guideline-based KPIs with a diverse stakeholder group, including those with lived experience of recurrent miscarriage. Insights into our process experiences may also help others undertaking similar projects to develop guideline-based KPIs, particularly those undertaken in the absence of a clinical guideline, and/or which involve a range of stakeholders.

## Supplementary Information

**Additional file 1.** GRIPP2-SF Reporting Checklist.

**Additional file 2.** Sample item, Delphi survey—Round 1.

**Additional file 3.** Sample item, Delphi survey—Round 2.

**Additional file 4.** Sample item, Consensus meetings.

**Additional file 5.** Sample item, Final survey.

**Additional file 6.** Overview of item rating during e-Delphi survey, by stakeholder category, recommendation/outcome category and round.

**Additional file 7.** List of KPIs not retained.

**Additional file 8.** Overview of item rating during final survey, by stakeholder category, KPI category.

## Data Availability

The datasets used and/or analysed during the current study are available from the corresponding author on reasonable request.

## Abbreviations

AGREE II: Appraisal of Guidelines for Research and Evaluation
KPI: Key performance indicator
RRAG: RE:CURRENT Research Advisory Group
