# Publisher Correction to: Developing guideline-based key performance indicators for recurrent miscarriage care: lessons from a multi-stage consensus process with a diverse stakeholder group

**DOI:** 10.1186/s40900-022-00368-4

**Published:** 2022-08-02

**Authors:** Marita Hennessy, Laura Linehan, Rebecca Dennehy, Declan Devane, Rachel Rice, Sarah Meaney, Keelin O’Donoghue

**Affiliations:** 1 Pregnancy Loss Research Group, Department of Obstetrics and Gynaecology, University College Cork, Cork, T12 DC4A Ireland; 2 INFANT Research Centre, University College Cork, Cork University Maternity Hospital, Cork, T12 DC4A Ireland; 3 College of Medicine and Health, University College Cork, Cork, T12 EKDO Ireland; 4 School of Nursing and Midwifery, National University of Ireland, Galway, Galway, H91 E3YV Ireland; 5 Evidence Synthesis Ireland, National University of Ireland, Galway, Galway, H91 E3YV Ireland; 6 School of Applied Social Studies, University College Cork, Cork, T12 D726 Ireland; 7 National Perinatal Epidemiology Centre, University College Cork, Cork University Maternity Hospital, Cork, T12 DC4A Ireland

## Correction to: Research Involvement and Engagement (2022) 8:18 10.1186/s40900-022-00355-9

Following publication of the original article [1], the authors reported errors in the Feedback from participants on the KPI development process section. The revised Feedback from participants on the KPI development process section is indicated hereafter and the changes have been highlighted in **bold typeface**.

The correct Feedback from participants on the KPI development process section should read:

**Feedback from participants on the KPI development process**

The word cloud generated from participants’ feedback on the KPI development process is presented in Fig. 3.

Words that predominated related to the long, complicated, time-consuming process; despite this, positives were noted relating to comprehensiveness, good facilitation, learning, and engagement/participation.

These findings were further elaborated on in participants’ responses to the questions posed around what worked well and what could be done differently; participant quotes are designated by identifiers W(ell) and B(etter), relating to the latter. We generated three themes: accessibility, richness in diversity, streamlining the development process.

*Richness in diversity* describes the benefits stated by some participants, including multiple/diverse perspectives, the rich discussions, learning (which they may have missed out on if they knew the time commitment involved initially; they would have ‘baulked at the outset’), and how it ‘gives the project a lot of weight’.

“The discussions which flowed during the meetings were brilliant, and the knowledge and passion of the people on the group is inspirational. I am delighted to be part of the group” (W3)

**Within this theme, some areas for improvement were noted, such as the benefit of having more representation from doctors/midwives in training and more**

“alternative voices to the medical expertise that was on the group but were equally informed in terms of fertility, miscarriage” (B7).

*Accessibility* represents the majority of comments received and describes what facilitated participants to access/engage with the process, or not. Sub-themes encompassed: skilled facilitation, communication with/from the research team, virtual access/timing of meetings, and making the process more user-friendly. Participants valued the skilled facilitation during the consensus meetings, lay explanations provided, and adequate time for discussion.

“Some of the consensus meetings were……….heavy and I sometimes felt I was overwhelmed with all the medical jargon, but Keelin’s [KOD’s] explanations were super as well as Declan’s [DD’s] and other members in the group” (W5)

**Some thought that providing a reference guide with an explanation of medical terms at the outset would have been very beneficial. One parent advocate felt that**

“the responsibility felt heavy at times, to raise questions from a parent perspective without the medical expertise”

**and suggested that it would have been helpful for the parent representatives to have met together with the team, prior to starting the process to consider their role in it and to strengthen their voice perhaps (B13). A few participants stated that the time commitment involved should have been made clearer at the outset.**

Participants highlighted positives regarding the responsiveness/accessibility of research team members (email/phone communication) when information/clarification was needed, updates regarding progress and information in advance, and honesty around the challenges experienced during the development process. Many felt that the virtual format, and evening meetings, facilitated access; the shorter (3-h) meetings, rather than one long day, were generally preferred. The sub-theme ‘making the process more user-friendly’ related to comments from a few participants about the difficultly experienced with the Delphi survey, including the inability to ‘save and continue later’ on the online platform, as well as one noting that the ability to abstain from voting during consensus meeting should have been clearer at the outset.

*Streamlining the development process* captures comments made by a few participants about how the number of recommendations/KPIs could have been narrowed down—by those with the relevant expertise— before asking all participants to vote on them.

“Perhaps the KPIs could have been narrowed down by those who really had the expertise to do that prior to the big group coming together to vote on them—or else, depending on people’s backgrounds, being invited to come for voting on sections that were only within people’s expertise/experience.” (B16)

All the changes requested are implemented in this Publisher correction and the original article [1] has been corrected. The publisher apologises to the authors and readers for the inconvenience caused by this mistake.

## References

[1] Hennessy M, Linehan L, Dennehy R (2022). Developing guideline-based key performance indicators for recurrent miscarriage care: lessons from a multi-stage consensus process with a diverse stakeholder group. Res Involv Engagem.

