# Supplementary material for: Developing guideline-based key performance indicators for recurrent miscarriage care: lessons from a multi-stage consensus process with a diverse stakeholder group
Source: Res Involv Engagem. 2022 May 14;8:18. doi: 10.1186/s40900-022-00355-9 (PMC9107009; doi:10.1186/s40900-022-00355-9)
Supplement: Supplementary file 1 — Additional file 1. GRIPP2-SF Reporting Checklist. [file 40900_2022_355_MOESM1_ESM.docx]

**Additional File 1 GRIPP2-SF Reporting Checklist**

| **Section and topic** | **Item** | **Reported on page no.** |
| --- | --- | --- |
| 1: Aim | Report the aim of PPI in the study | 2,6 |
| 2: Methods | Provide a clear description of the methods used for PPI in the study | 7-17 |
| 3: Study results | Outcomes—Report the results of PPI in the study, including both positive and negative outcomes | 17-26 |
| 4: Discussion and conclusions | Outcomes—Comment on the extent to which PPI influenced the study overall. Describe positive and negative effects | 27-33 |
| 5: Reflections/critical perspective | Comment critically on the study, reflecting on the things that went well and those that did not, so others can learn from this experience | 27-33 |
