# Supplementary figures and images for: Developing guideline-based key performance indicators for recurrent miscarriage care: lessons from a multi-stage consensus process with a diverse stakeholder group
Source: Res Involv Engagem. 2022 May 14;8:18. doi: 10.1186/s40900-022-00355-9 (PMC9107009; doi:10.1186/s40900-022-00355-9)

**Additional File 2 Sample item, Delphi survey – Round 1**


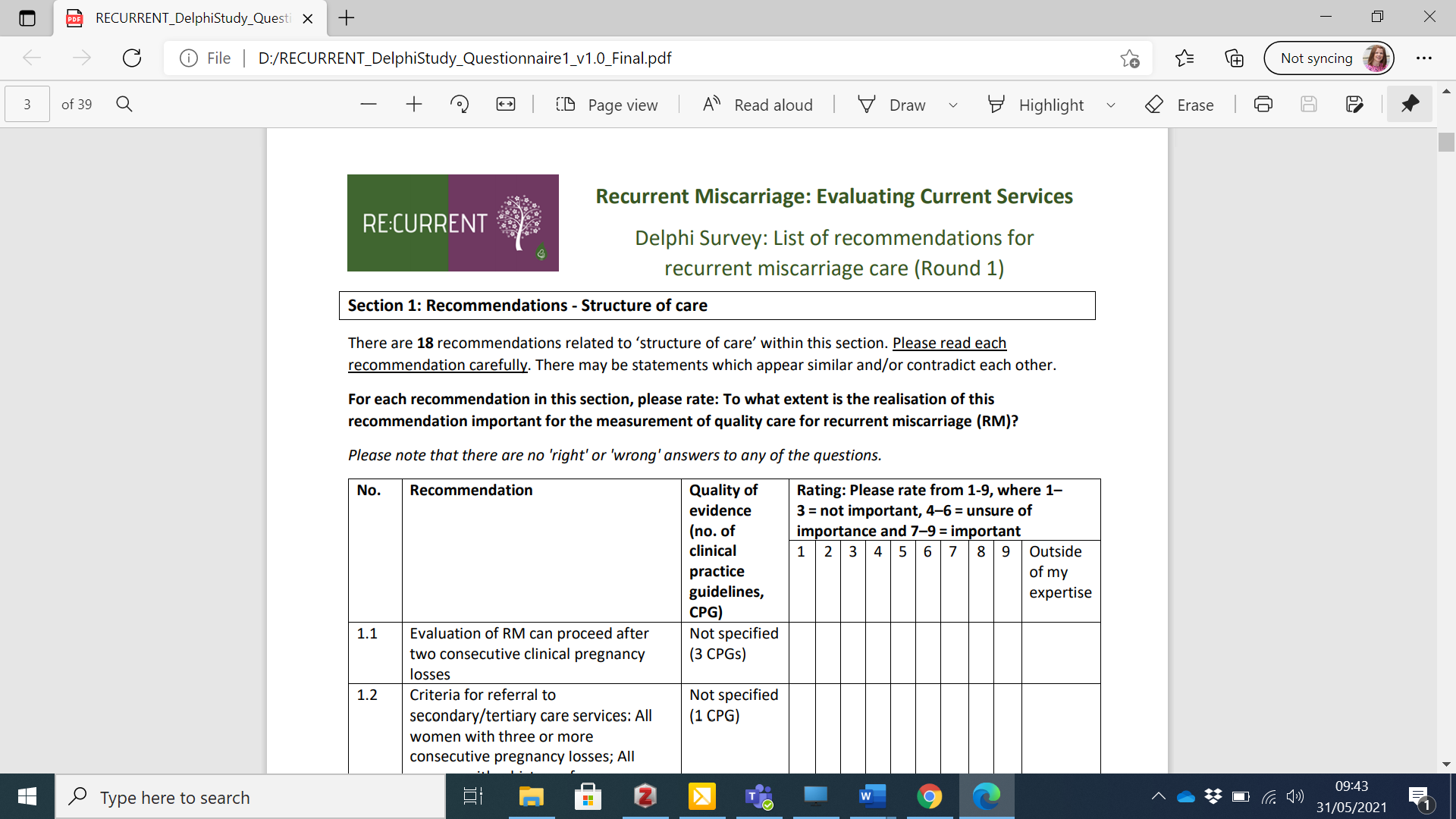

Supplement: Supplementary file 2 — Additional file 2. Sample item, Delphi survey—Round 1. [file 40900_2022_355_MOESM2_ESM.docx]

**Additional File 3 Sample item, Delphi survey – Round 2**


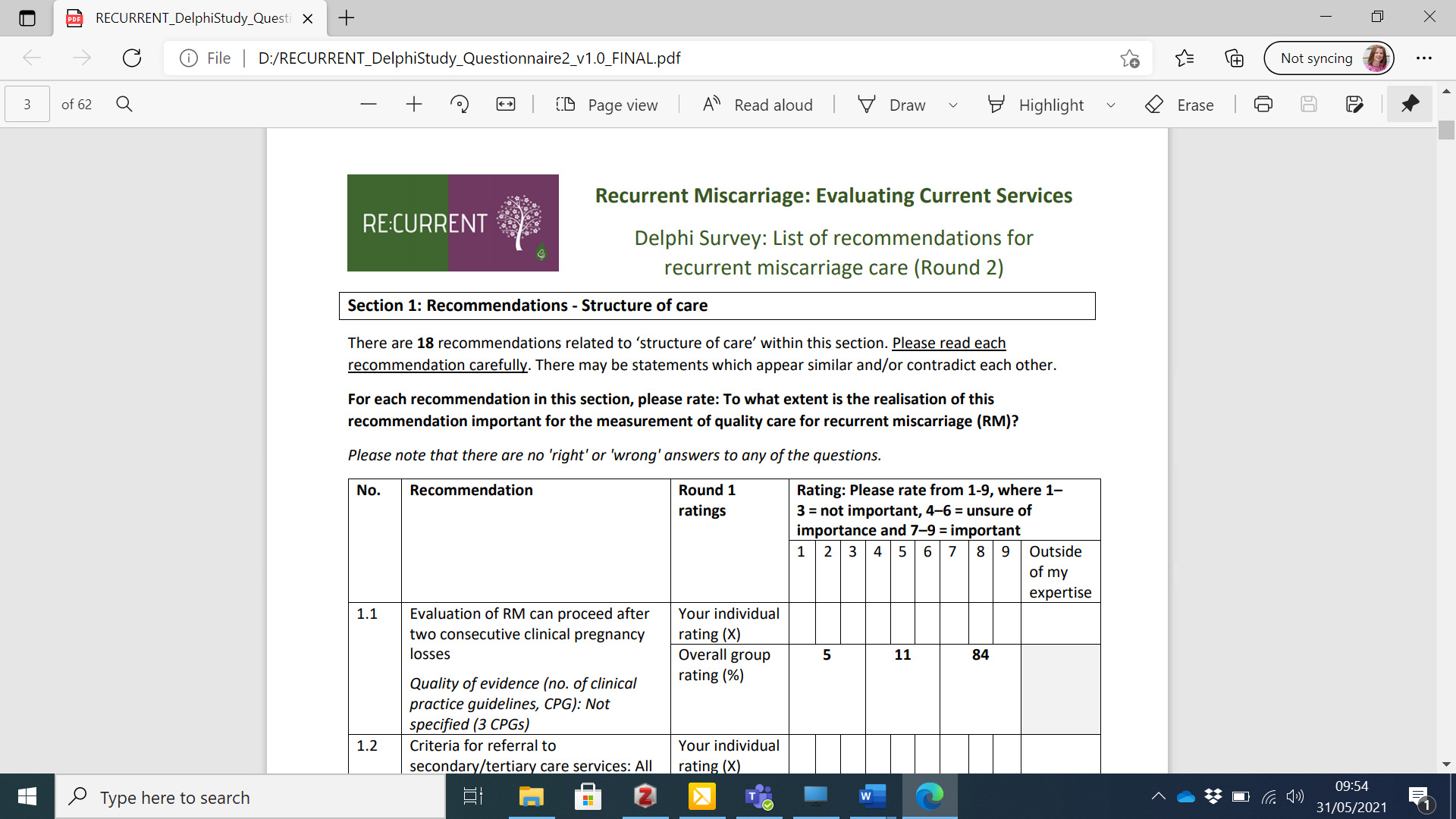

Supplement: Supplementary file 3 — Additional file 3. Sample item, Delphi survey—Round 2. [file 40900_2022_355_MOESM3_ESM.docx]

**Additional File 4 Sample item, Consensus meetings**


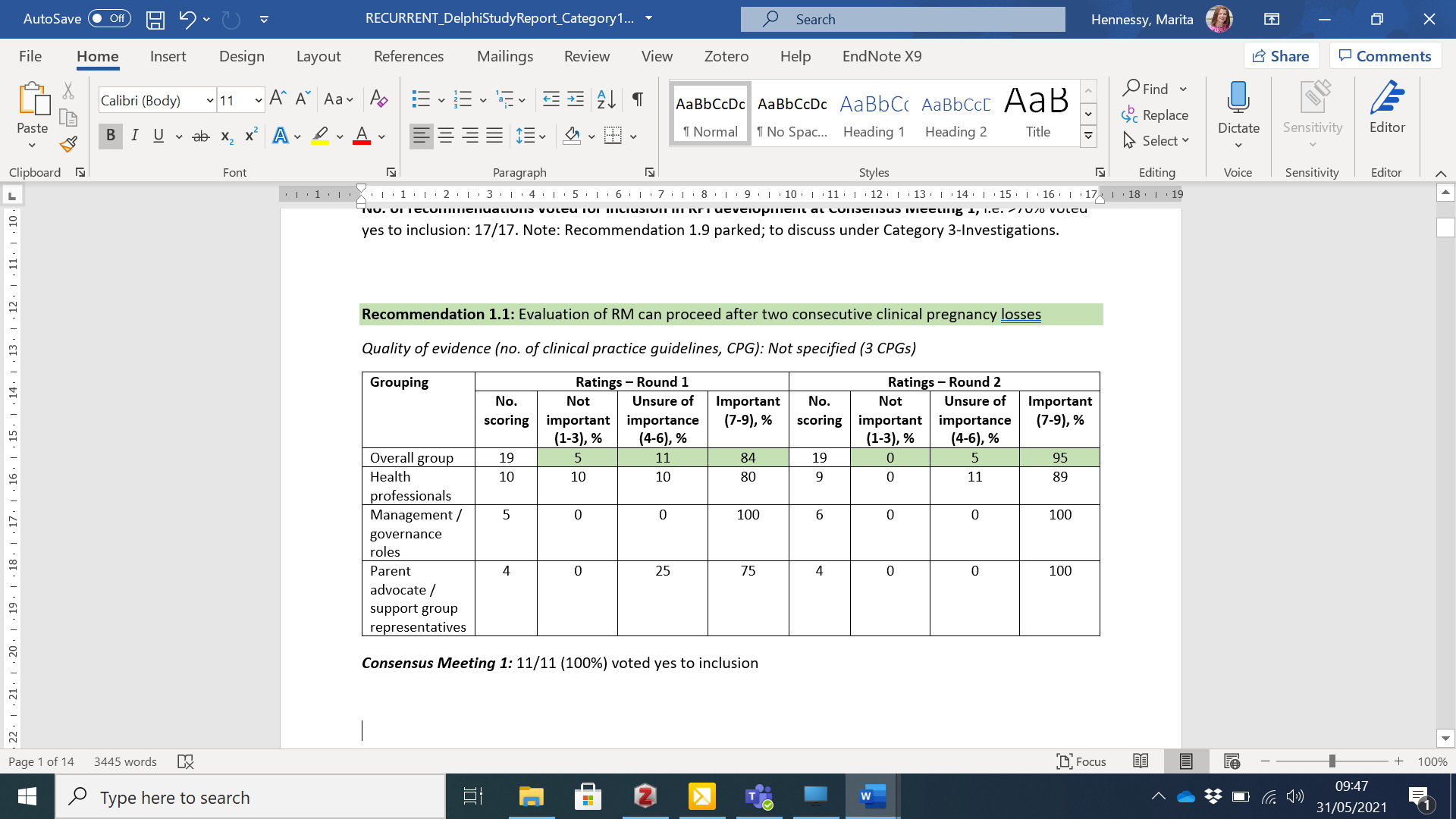

Supplement: Supplementary file 4 — Additional file 4. Sample item, Consensus meetings. [file 40900_2022_355_MOESM4_ESM.docx]

**Additional File 5 Sample item, Final survey**


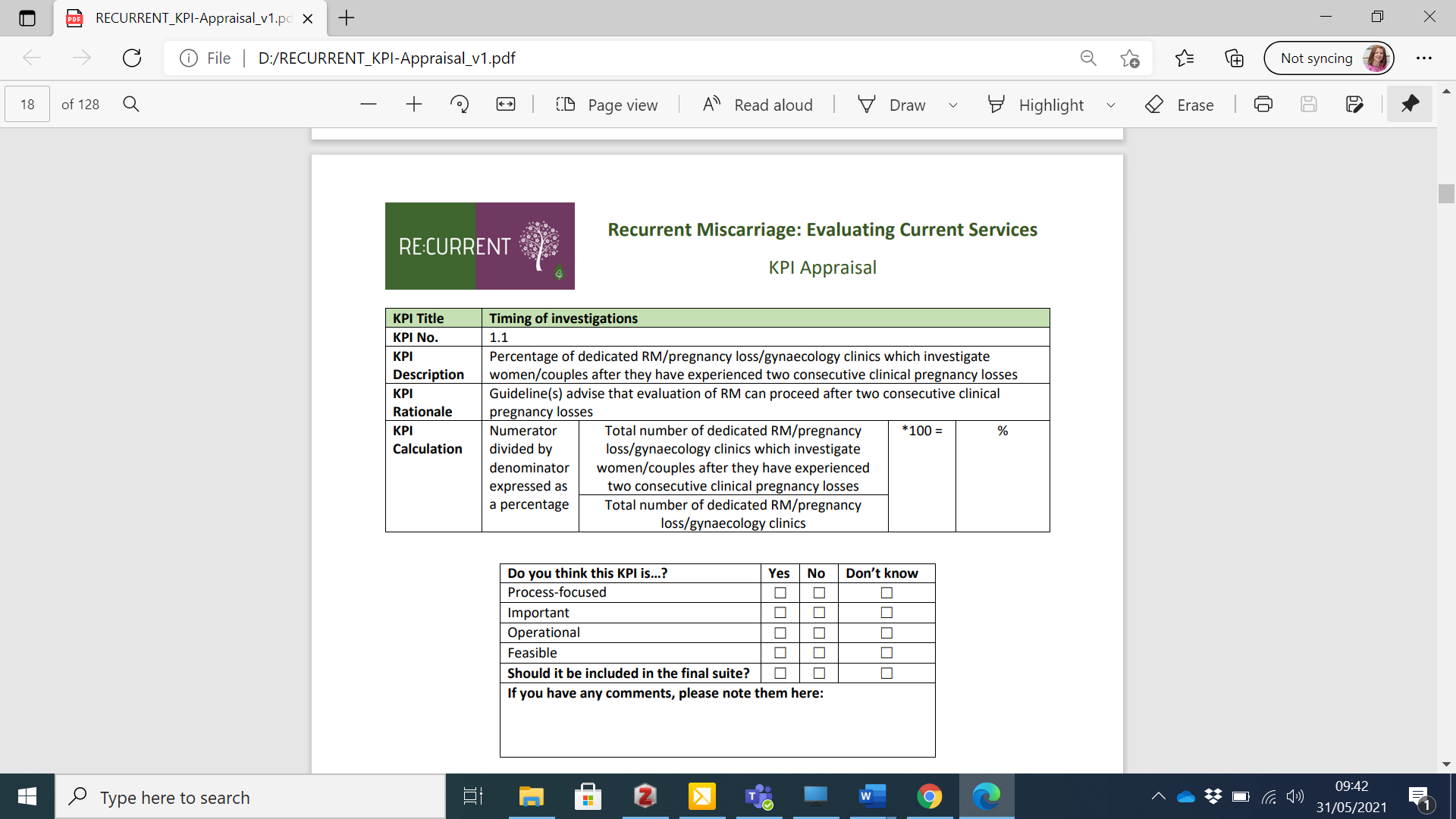

Supplement: Supplementary file 5 — Additional file 5. Sample item, Final survey. [file 40900_2022_355_MOESM5_ESM.docx]
