# Supplementary material for: Developing guideline-based key performance indicators for recurrent miscarriage care: lessons from a multi-stage consensus process with a diverse stakeholder group
Source: Res Involv Engagem. 2022 May 14;8:18. doi: 10.1186/s40900-022-00355-9 (PMC9107009; doi:10.1186/s40900-022-00355-9)
Supplement: Supplementary file 6 — Additional file 6. Overview of item rating during e-Delphi survey, by stakeholder category, recommendation/outcome category and round. [file 40900_2022_355_MOESM6_ESM.docx]

**Additional File 6 Overview of item rating during e-Delphi survey, by stakeholder category, recommendation/outcome category and round**

| **Stakeholder category** | **Structure of care** | | **Counselling / supportive care** | | **Investigations** | | **Treatment** | | **Outcomes** | |
| --- | --- | --- | --- | --- | --- | --- | --- | --- | --- | --- |
|  | **R1** | **R2** | **R1** | **R2** | **R1** | **R2** | **R1** | **R2** | **R1** | **R2** |
| **Health professionals** | | | | | | | | | | |
| Total no. of participants | 10 | 9 | 10 | 9 | 10 | 9 | 10 | 9 | 10 | 9 |
| Total no. of items in this category | 18 | 18 | 13 | 13 | 76 | 76 | 80 | 80 | 14 | 14 |
| Overall no. of items voted on by participants / total | 180 / 180 | 162 / 162 | 128 / 130 | 117 / 117 | 649 / 760 | 619 / 684 | 683 / 800 | 604 / 720 | 140 / 140 | 126 / 126 |
| Average no. of items assigned votes by each participant | 18 | 18 | 12.8 | 13 | 64.9 | 68.8 | 68.3 | 67.1 | 14 | 14 |
| Range | N/A | N/A | 12-13 | N/A | 23-76 | 39-76 | 3-80 | 11-80 | N/A | N/A |
| **Management / governance role** | | | | | | | | | | |
| Total no. of participants | 6 | 6 | 6 | 6 | 6 | 6 | 6 | 6 | 6 | 6 |
| Total no. of items in this category | 18 | 18 | 13 | 13 | 76 | 76 | 80 | 80 | 14 | 14 |
| Overall no. of items voted on by participants / total | 101 / 108 | 108 / 108 | 74 / 78 | 78 / 78 | 131 / 456 | 229 / 456 | 122 / 480 | 122 / 480 | 80 / 84 | 70 / 84 |
| Average no. of items assigned votes by each participant | 16.8 | 18 | 12.3 | 13 | 21.8 | 38.2 | 20.3 | 20.3 | 13.3 | 11.7 |
| Range | 13-18 | N/A | 10-13 | N/A | 2-70 | 9-70 | 0-64 | 0-64 | 12-14 | 0-14 |
| **Parent advocate / support group representative** | | | | | | | | | | |
| Total no. of participants | 4 | 4 | 4 | 4 | 4 | 4 | 4 | 4 | 4 | 4 |
| Total no. of items in this category | 18 | 18 | 13 | 13 | 76 | 76 | 80 | 80 | 14 | 14 |
| Overall no. of items voted on by participants / total | 71 / 72 | 71 / 72 | 46 / 52 | 48 / 52 | 114 / 304 | 124 / 304 | 86 / 320 | 112 / 320 | 52 / 56 | 56 / 56 |
| Average no. of items assigned votes by each participant | 17.8 | 17.8 | 11.5 | 12 | 28.5 | 31 | 21.5 | 28 | 13 | 14 |
| Range | 17-18 | 17-18 | 10-13 | 11-13 | 1-63 | 1-65 | 0-48 | 0-60 | 11-14 | N/A |

Note: R=Round
