# Supplementary material for: Developing guideline-based key performance indicators for recurrent miscarriage care: lessons from a multi-stage consensus process with a diverse stakeholder group
Source: Res Involv Engagem. 2022 May 14;8:18. doi: 10.1186/s40900-022-00355-9 (PMC9107009; doi:10.1186/s40900-022-00355-9)
Supplement: Supplementary file 7 — Additional file 7. List of KPIs not retained. [file 40900_2022_355_MOESM7_ESM.docx]

**Additional File 7 List of KPIs not retained**

| **KPI No.** | **KPI Title** | **KPI Sub-category** |
| --- | --- | --- |
| **KPI category: Investigations (n=5)** | | |
| 3.9 | Assessment of uterine anatomy – use of MRI | Anatomical investigations |
| 3.17 | Human leukocyte antigen (HLA) determination | Immunological screening |
| 3.18 | Cytokine testing | Immunological screening |
| 3.19 | Measurement of anti-Hy antibodies | Immunological screening |
| 3.39 | Prolactin testing | Metabolic & endocrinologic factors |
| **KPI category: Treatment (n=2)** | | |
| 4.32 | Treatment of euthyroid women with thyroid antibodies | Recurrent miscarriage with metabolic and endocrinologic factors |
| 4.24 | Antioxidants for men | Recurrent miscarriage with male factor |
