# Supplementary material for: Developing guideline-based key performance indicators for recurrent miscarriage care: lessons from a multi-stage consensus process with a diverse stakeholder group
Source: Res Involv Engagem. 2022 May 14;8:18. doi: 10.1186/s40900-022-00355-9 (PMC9107009; doi:10.1186/s40900-022-00355-9)
Supplement: Supplementary file 8 — Additional file 8. Overview of item rating during final survey, by stakeholder category, KPI category. [file 40900_2022_355_MOESM8_ESM.docx]

**Additional File 8 Overview of item rating during final survey, by stakeholder category, KPI category**

| **Stakeholder category** | **Structure of care** | **Counselling / supportive care** | **Investigations** | **Treatment** | **Outcomes** |
| --- | --- | --- | --- | --- | --- |
| **Health professionals** | | | | | |
| Total no. of participants | 7 | 7 | 7 | 7 | 7 |
| Total no. of items in this category | 20 | 7 | 35 | 36 | 19 |
| Overall no. of items voted on by participants / total | 133/140 | 40/49 | 219/245 | 222/252 | 132/133 |
| Average no. of items assigned votes by each participant | 19 | 5.7 | 31.3 | 31.7 | 18.9 |
| Range | 13-20 | 4-7 | 19-35 | 9-36 | 18-19 |
| **Management / governance role** | | | | | |
| Total no. of participants | 4 | 4 | 4 | 4 | 4 |
| Total no. of items in this category | 20 | 7 | 35 | 36 | 19 |
| Overall no. of items voted on by participants / total | 69/80 | 23/28 | 57/140 | 42/144 | 51/76 |
| Average no. of items assigned votes by each participant | 17.3 | 5.8 | 14.3 | 10.5 | 12.8 |
| Range | 10-20 | 2-7 | 3-21 | 5-15 | 1-19 |
| **Parent advocate / support group representative** | | | | | |
| Total no. of participants | 3 | 3 | 3 | 3 | 3 |
| Total no. of items in this category | 20 | 7 | 35 | 36 | 19 |
| Overall no. of items voted on by participants / total | 59/60 | 18/21 | 87/105 | 76/108 | 54/57 |
| Average no. of items assigned votes by each participant | 19.7 | 6 | 29 | 25.3 | 18 |
| Range | 19-20 | 5-7 | 27-31 | 19-31 | 16-19 |
